# Unveiling the Diagnostic and Prognostic Value of Inflammatory Cytokines in Preeclampsia: A Review of Ιnterleukins IL-15, IL-16, IL-17 and IL-35

**DOI:** 10.3390/jcm14238322

**Published:** 2025-11-23

**Authors:** Arzou B. Chalil, Emmanouil Andreou, Paraskevas Perros, Ioakeim Sapantzoglou, Antonios Koutras, Ioannis Prokopakis, Dionysios Metaxas, Athanasios Chionis, Georgios Tsakaldimis, Nikoletta Koutlaki, Christina Tsigalou, Emmanuel N. Kontomanolis

**Affiliations:** 1 Department of Obstetrics and Gynecology, Democritus University of Thrace, 68100 Alexandroupolis, Greece; arzou.m.chalil@gmail.com (A.B.C.); nikoleta_koutlaki@yahoo.gr (N.K.); mek-2@otenet.gr (E.N.K.); 2 Department of Gynecology, Laiko General Hospital of Athens, 11527 Athens, Greece; paris_per@yahoo.gr (P.P.); ath.chionis@yahoo.gr (A.C.); 3 1st Department of Obstetrics and Gynecology, National and Kapodistrian University of Athens, General Hospital of Athens ‘ALEXANDRA’, 11528 Athens, Greece; kimsap1990@hotmail.com; 4 The Fertility Center, Chelsea and Westminster Hospital NHS Foundation Trust, Imperial College, London SW10 9NH, UK; antoniskoy@yahoo.gr; 5 Bourn Hall, Fertility Clinc, Cambridge CB23 2TN, UK; ioannisprokopakis@gmail.com; 6 Department of Internal Medicine, Erythros Stavros Hospital, 11526 Athens, Greece; dionmetaxas@hotmail.com; 7 Department of Urology, Democritus University of Thrace, 68100 Alexandroupolis, Greece; g.tsalkadimis@hotmail.com; 8 Laboratory of Hygiene and Environmental Protection, Faculty of Medicine, Democritus University of Thrace, 68100 Alexandroupolis, Greece; ctsigalo@med.duth.gr

**Keywords:** preeclampsia (PE), pregnancy, interleukin (IL)-15, IL-16, IL-17, IL-35, inflammatory response

## Abstract

**Background:** Preeclampsia (PE) is a multicomplex disorder occurring during pregnancy, characterized by the onset of hypertension and proteinuria, or hypertension accompanied by organ dysfunction (such as impaired liver function, renal insufficiency, pulmonary edema, or cerebral and visual impairment), with or without proteinuria in the latter half of pregnancy or postpartum. It impacts around 5% of all pregnancies, resulting in considerable fetal and maternal mortality and morbidity. A properly regulated inflammatory response is crucial for achieving a successful pregnancy; nevertheless, an excessive reaction appears to contribute to the onset of this syndrome. This review sought to investigate the role and correlation of interleukins (IL)-15, IL-16, IL-17, and IL-35 in the pathogenesis of PE, along with the prospective application of biomarkers in predicting and monitoring this illness. **Methods:** A thorough investigation was performed in the PubMed/Medline, Scopus, and Google Scholar electronic databases up to September 2025, employing the terms PE, Pregnancy, IL-15, IL-16, IL-17, IL-35, Inflammatory Response, and Cytokines. Women at the time of diagnosis were matched with normotensive counterparts of similar gestational age. **Results:** A total of 30 full-text articles were obtained following a thorough assessment. The majority of the published data showed that women with preeclampsia have significantly higher levels of IL-15 and IL-17 in their plasma compared to normotensive women, whereas IL-35 levels were mainly decreased, respectively. Moreover, IL-16 levels were elevated across all the studies, primarily correlating with condition severity. **Conclusions:** Collectively, IL-15, IL-16, IL-17, and IL-35 are markedly linked to the immunopathology of preeclampsia, with elevated maternal serum levels corresponding with the presence and severity of the disease. These cytokines demonstrate potential as biomarkers for diagnosis, prognosis, and disease surveillance. Future study, including the examination of cytokine profiles in placental and amniotic fluid, as well as additional intriguing cytokines, is essential to clarify their prognostic importance and mechanistic roles.

## 1. Introduction

A healthy pregnancy is characterized by a regulated inflammatory process. A proinflammatory environment is crucial for effective implantation and parturition. Nevertheless, the semi-allogenic fetus, in order to have a proper placentation and development during pregnancy, requires a tolerogenic environment [1]. This condition is intensified in preeclampsia (PE) due to excessive placental stimuli [2].

PE is a complex, progressive multisystem hypertensive disorder during pregnancy, characterized by the combination of new-onset hypertension and proteinuria or other organ dysfunction (impaired liver function, development of renal insufficiency, pulmonary edema, cerebral or visual impairment), with or without proteinuria in the second half of pregnancy or postpartum [3,4]. It is essential to emphasize that PE has a detrimental impact on both the mother and the fetus. The International Federation of Gynaecology and Obstetrics (FIGO) has adopted the definition of the International Society for the Study of Hypertension in Pregnancy (ISSHP), where PE is defined as gestational hypertension accompanied by either proteinuria (i.e., >30 mg/mol protein; creatinine ratio; 300 mg/24 h) or other maternal organ dysfunction. These conditions include acute kidney injury (AKI), liver dysfunction, neurological complications, or hematological complications. Creatinine levels above 90 μmol/L, 1 mg/dL, set the diagnosis of AKI. Liver dysfunction is characterized by elevated transaminases, such as alanine aminotransferase or aspartate aminotransferase (>40 IU/L). In addition, hematological issues encompass thrombocytopenia, meaning platelet count < 150000/μL, disseminated intravascular coagulation, or hemolysis. PE results in the placental dysfunction that is manifested with fetal growth restriction (FGR), abnormal umbilical artery Doppler waveforms, or even stillbirths [5,6].

The prevalence of PE is assumed to be between 3 and 5% of all pregnancies [7,8,9].

The Figo classification defines early-onset PE as PE occurring before 34 weeks of gestation, while late-onset PE is characterized by a first diagnosis set at 34 weeks of gestation or later.

The underlying mechanisms are not fully understood; however, genetic, maternal factors (such as chronic hypertension, diabetes, and antiphospholipid antibodies) and immunological factors (including decidual natural killer cells and regulatory T cell imbalance) contribute to inadequate trophoblast invasion, resulting in shallow placentation (first phase or stage I).

The significance of cytokines in a successful pregnancy has been emphasized in a multitude of studies. Women with preeclampsia were found to exhibit increased concentrations of interleukin (IL)-1, IL-6, and tumor necrosis factor alpha (TNF-α) in their plasma and amniotic fluid, alongside elevated levels of IL-2 and interferon gamma (IFN-γ). All these inflammatory cytokines appear to adversely affect pregnancy development [10,11]. For instance, IL-6 is a versatile proinflammatory cytokine synthesized by activated vascular endothelial cells and the placenta. Elevated levels of IL-6 in maternal serum are associated with a more severe and earlier onset of preeclampsia [12].

The necessity of investigating potential biomarkers to predict this PE is extremely essential, as the only effective therapy for this condition remains the delivery of the fetus and placenta.

Peripheral blood mononuclear cells represent a potential source of inflammatory cytokines in the context of PE, which features angiogenic and anti-angiogenic abnormalities. When stimulated by extracellular vesicles from syncytiotrophoblast, isolated from the placental explants of women with early PE, these cells can release cytokines such as IL-6, TNF-α, IL-8, IL-10, IL-17, granulocyte colony-stimulating factor, macrophage inflammatory protein (MIP)-1α, and MIP-1β [13].

IL-15 was initially identified as a factor that activates T-lymphocytes and shares structural similarities with IL-2, whereas IL-16 initially was described as a T-lymphocyte chemoattractant that utilizes CD4 as its receptor. Both are pleiotropic cytokines. IL-15 induces T cell proliferation and B cell maturation, and it is especially essential for NK cell development and cytotoxicity [14]. IL-16 stimulates the production of proinflammatory cytokines such as IL-6, TNF-α, IL-1 alpha, and IL-15 by monocytes, and upregulation of IL-2 receptor alpha on T cells [15,16]. The expression of IL-15 and IL-16 by non-immune cells implies that they may have activities that extend beyond the immune system. Their presence at the maternal–fetal interface implies their role in pregnancy [15,16].

IL-17 is an inflammatory cytokine that is found to be secreted by Th17 cells and has been demonstrated to contribute to the progression of numerous inflammatory processes. Published data reported that human IL-17 was first identified in CD4+ T cells, then subsequently in CD8+ T cells, NKT cells, and monocytes. It is a dynamic contributor to the recruitment and activation of neutrophils, a notable characteristic of the inflammatory response observed in PE [17].

On the other hand, IL-35 is an immunosuppressive cytokine secreted by Treg cells, specifically produced by Foxp3+ regulatory T cells. Its structure consists of two chains: Ebi3 (IL-27β) and p35 (IL-12α) [18]. IL-35 has been identified as having three recognized biological effects: the inhibition of T cell proliferation, the transformation of naive T cells into IL-35-producing induced regulatory T cells (iTr35 cells), and the downregulation of Th17 cell development and differentiation, in addition to the regulation of autoimmune inflammation [19,20]. Ebi3 and p35, two components of IL-35, were discovered to be substantially co-expressed in placental trophoblasts [21].

This review aims to examine the correlation between the interleukins IL-15, IL-16, IL-17, and IL-35 in the pathogenesis of PE. Comprehending their role will augment their relevance as prospective biomarkers for both PE prediction and monitoring.

## 2. Material Methods

This review was systematically structured, designed, and carried out in compliance with the Preferred Reporting Items for Systematic Reviews and Meta-analyses (PRISMA) reporting guidelines. We thoroughly searched electronic databases: PubMed, Crossref, and Google Scholar, for papers issued up to September 2025, utilizing the search terms “PE”, “Pregnancy”, “IL-15”, “IL-16”, “IL-17”, “IL-35”, “Inflammatory Response”, and “Cytokines”. These keywords were also combined using Boolean operators (“OR” and “AND”). Subsequently, the references of the collected papers were examined to identify potentially relevant research. All records were meticulously examined, and duplicate entries were eliminated. Records screened according to the following inclusion and exclusion criteria.

Clinical trials, case reports, and cohort studies were all considered eligible to be analyzed and included in this review. The review consisted of studies that investigated the association of the maternal plasma levels of IL-15, IL-16, IL-17, and IL-35 with PE. The control group were normotensive pregnant women. The comparison with the corresponding gestational age control group was conducted at the time of diagnosis, that was, after 20 weeks of gestation and typically during the third trimester of pregnancy. No geographical restrictions were made.

Narrative and systematic reviews were excluded, along with animal studies and studies not conducted in English. Research assessing the levels of IL-15, IL-16, IL-17, and IL-35 in placenta or amniotic fluid was also omitted. Additionally, studies including multiple pregnancies or miscarriages caused by factors like autoimmune diseases, reproductive tract abnormalities, or chromosomal abnormalities were excluded.

Titles, summaries, and abstracts of all identified papers were scrutinized for study design, the nature of the association, and the final outcomes. Notably, two separate reviewers, P.P. and E.A., thoroughly reviewed and assessed 99 whole texts from relevant papers. If only one reviewer selected a study, the final choice was made by a third reviewer, A.K. Following meticulous assessment, 69 studies were removed due to inconsequential outcomes or limited sample sizes. Thirty research studies were designated for data extraction.

Data extraction was performed independently using a standardized form that encompassed the study design, sample size, participant characteristics, definitions and diagnostic criteria for preeclampsia, measured interleukins, assay methodologies, and principal findings (mean cytokine levels and statistical significance).

**Figure 1:**
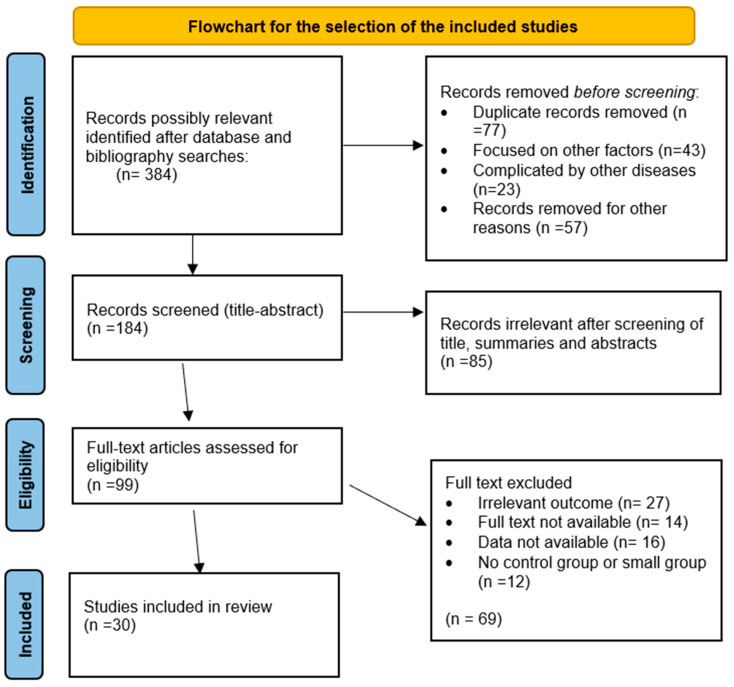
Flowchart for the selection of the included studies.

The Newcastle Ottawa Scale (NOS) is employed to assess the quality of the investigations, as illustrated in Table 1. A “√” is used to indicate that a study satisfies the criteria, while a “-” is used to indicate that it fails. The quality of the investigations was assessed as low (score 0–3), medium (score 4–6), and high (score 7–9). None of the studies received a score below 6. Figure 1 delineates the process for selecting the included studies, which are described in Table 2, Table 3, Table 4 and Table 5.

## 3. Results

A total of 184 citations were reviewed, and 30 studies met the eligibility criteria and were included in the final review. Among these, eight studies focused on IL-15 levels in women with PE, while eight studies examined IL-16 levels. Additionally, 19 studies reported IL-17 levels, and IL-35 was examined in 7 studies.

### 3.1. IL-15

Regarding the role of NK cells in the pathogenesis of PE, IL-15 could be a key player in the development of the disease. IL-15 is produced by human placental tissue culture, with blood levels correlating to the duration of pregnancy, peaking during the implantation phase in the decidua. In addition, IL-15 is crucial for NK cell growth and promotes the proliferation, cytokine production, and cytotoxicity of activated peripheral blood NK cells. Trophoblasts in the placenta can produce modest levels of IL-15 early in gestation but produce the most late in gestation during spontaneous labor.

Eight of the thirty studies focused on IL-15, including in total 880 pregnant women, of whom 402 suffered from PE and 478 were the controls. Table 2 summarizes the included studies. The majority of the studies revealed higher IL-15 levels in the PE group. Chaiworapongsa et al., who performed a case–control study including 96 PE women and 213 controls, found higher IL-15 levels or expression in the PE group versus the control group, highlighting that women with abnormal angiogenic profiles present worse clinical severity [26]. In alignment with them were the results reported by the observational studies conducted by Kalantar et al. and Mansouri et al., which took place in Iran [23,25]. Furthermore, El-Barabie et al. in Egypt and Hu et al. in China, in their own observational studies, reported higher levels in women who suffered from PE, especially in the group with the severe form of PE [22,24]. Nevertheless, Lu et al., Jonsson et al., and Martinez-Fierro et al. were the only studies reporting low or undetectable IL-15 expression or no significant difference in the second-trimester serum IL-15, respectively, between the studied groups [27,28,29]. Nonetheless, the limited sample size in the experiments conducted by Lu et al. and Jonsson et al. constitutes a constraint when compared to other research. In addition, this disagreement may arise from the fact that the authors observed a little, although non-significant, increase. An assumption could be that the proportion of mild instances was significantly larger than that of severe ones, leading to the authors’ inability to achieve statistically significant levels.

### 3.2. IL-16

IL-16 is a critical component of innate immune responses. However, there is a scarcity of information regarding the function of IL-16 in human pregnancy. Regarding the role of IL-16, the findings were consistent across all the studies evaluating IL-16, showing significantly higher levels in PE. Eight studies, which are summarized in Table 3, were focused on the role of IL-16, including a total of 989 pregnant women; 510 were in the PE group and 479 were the normotensive ones. Specifically, El-Barabie et al. and Hu et al., in their observational studies in Egypt and China, respectively, found elevated IL-16 levels in severe PE compared to mild PE [22,24]. Moreover, another retrospective study from China, by Lu et al., who compared 40 PE women with 40 controls, revealed higher levels of IL-16 in the studied groups and additionally observed higher levels in the late-onset PE [27]. These results were in alignment with Radulescu’s et al. prospective study, performed in the period 2014–2015, which revealed significant increased levels of IL-16 in women who suffered from PE. The authors observed significantly higher IL-16 levels also in the those who developed severe PE [30]. These results were confirmed by two more studies that came from the USA, by Chaiworapongsa et al. and Gu et al. [26,31]. One important discovery in Gu et al.’s study was that, in comparison to normal pregnant controls (*n* = 63), IL-16 levels were significantly higher in severe PE (*n* = 51) and elevated in moderate PE (*n* = 11), with values of 287 ± 46 and 515 ± 58 (*p* < 0.01) versus 163 ± 9 pg/mL, respectively. In cases of severe PE, IL-16 levels were significantly higher than those observed in moderate PE (*p* < 0.05) and in gestationally matched normal pregnant controls (*p* < 0.01). Elevated IL-16 levels in PE are correlated with disease severity, according to the data [31]. Another study, also conducted by Li W et al., who investigated 131 PE women and 65 normotensive women, revealed significantly elevated levels of IL-16 [32]. Last but not least, the observational study by Wang et al., which consisted of 34 PE and 27 controls, reported higher maternal plasma levels of IL-16 in the PE group [33].

### 3.3. IL-35

IL-35 is produced and secreted by human trophoblast cells during the first trimester, which may contribute to the immunosuppressive properties of maternal immune cells during normal gestation. IL-35 may be a critical component of the cytokine network that regulates local immune responses during human gestation [52]. Seven out of thirty studies focused on IL-35 expression in PE, including a total of 633 pregnant women (PE group = 354, Control = 279). Table 4 consolidates the included studies.

Cao et al. and Li et al., two observational studies performed in China and published in 2015 and 2020, respectively, reported lower levels of IL-35 in PE women compared to controls [36,51]. Notably, Cao et al. further demonstrated a dose-dependent decrease in IL-35 with increasing PE severity. This is also consistent with an observational study from China by Lu et al., who compared 90 PE women with 45 controls [35]. Furthermore, Ozkan’s et al. case–control study was consistent with the previous studies. The authors compared 40 PE women with 40 healthy pregnant ones and observed significantly lower levels of IL-35 in the PE group [38]. Decreased levels were also reported in another observational study by Cao et al. in 2018 [37]. Nevertheless, whereas most of the studies indicated lower levels of IL-35 in the serum of PE women, the observational study conducted in Iran by Batebi et al., comprising 100 PE women and 100 controls, reported higher levels of IL-35 in the PE compared to the controls [40]. In advance, a case–control study by Agha et al. spotted no difference between the PE group and the controls [50]. The discordance in the results claimed by Batebi et al., the only observational study that came from Iran, could be attributed to variations in Treg activity between populations or differences in inflammatory burden.

### 3.4. IL-17

Sixteen out of thirty studies focused on the presence of IL-17 in women with PE, which are outlined in Table 5. The majority of the studies that measured IL-17 maternal plasma levels in the PE group reported consistently higher IL-17 compared to the control groups. In total, 1762 pregnant women were investigated, with 883 of them in the PE group and 879 classified as the control group.

Molvarec et al. performed an observational study during 2013–2015, when they measured IL-17 in 59 PE women and in 60 healthy pregnant ones. They concluded that the levels of IL-17A were increased in the PE group. At the same time, the authors observed no correlation between IL-17A and sFlt-1, PIGF, or the sFlt-1/PIGF ratio. IL-17A and the sFlt-1/PIGF ratio had an additive effect on PE risk [34]. In alignment with them is the observational study by Lu et al. during the period 2015–2017 [35]. In addition, Cao et al. (2015 and 2018) reported IL-17 levels significantly higher in the PE group compared to the control group, but no difference between mild and severe PE [36,37]. The authors observed, also, a positive correlation between IL-17 levels and BMI, as well as between IL-17 levels and proteinuria. Additionally, Chaiworapongsa et al. highlighted increased IL-17 specifically in patients with abnormal angiogenic profiles [26]. Furthermore, the case–control study conducted by El Shahaway et al. demonstrated that the mean value of IL-17 in the PE group was 18.5 pg/mL, while in the control group it was 4.3 pg/mL. This discrepancy was statistically significant between the two groups [39]. Although Batebi et al. reported no difference between mild and severe PE, IL-17 levels were still significantly higher in PE overall [40]. Consistent with the above is also the observational study by Poordast T et al., comprising 30 PE women and 30 healthy ones [41]. Another study by Darmochwal-Kolarz D et al., (2012) reported higher levels of IL-17A in the serum of PE women compared to the normotensive ones [42]. In addition, two observational studies from China reported significant elevated levels of IL-17 in women who suffered from PE [44,45]. Lang et al. from China reported also significant elevated levels of IL-17A in the PE group compared to healthy pregnant women [46]. A case–control study performed in Brazil, by Perucci et al., involving 24 severe PE women and 34 healthy pregnant controls reported increased levels of IL-17A [47]. In alignment with them, another two case–control studies, one by Chen et al. and the other by Toldi et al., who compared PE women with normotensive ones, ended up with statistically elevated levels of IL-17 in the PE group [48,49]. Elevated levels were stated, in addition, in the case–control study performed by Agha et al. [50]. No statistical difference was spotted in the case–control study by Jonsson et al. [29]. On the other hand, the case–control study by Ozkan ZS et al., who compared 40 PE women to 40 healthy ones, reported significantly lower levels in the PE group of IL-17 [38].

## 4. Discussion

The relationship between excessive inflammatory response and preeclampsia is an area of intense research interest with great potential for understanding the pathophysiology of this condition.

A healthy pregnancy is a paradigm of immune tolerance [20]. Exaggerated intravascular inflammation is a hallmark that characterizes women with early preeclampsia; however, the involvement of inflammation in the pathophysiology of late preeclampsia is still being explored [53,54]. A recent comprehensive review indicates that most studies found women with preeclampsia exhibit elevated blood levels of cytokines (IL-6, IL-8, TNF-α, and C-reactive protein) compared to normotensive pregnant women [55]. The behavior of other cytokines, such as IL-10, was also reported to be elevated among studies [26].

A meta-analysis performed by Guan et al. involving 2549 participants revealed that patients with PE had notably higher levels of C-reactive protein (CRP), interleukins (IL)-4, IL-6, IL-8, IL-10, and tumor necrosis factor (TNF) in comparison to the controls. Concentrations of CRP and proinflammatory cytokines surpassed those of anti-inflammatory cytokines. Patients with a gestational age exceeding 34 weeks demonstrated significantly increased IL-6 and TNF levels. Those with higher systolic blood pressure showed elevated IL-8, IL-10, and CRP levels [56]. The significance of the increased level of inflammatory cytokines like TNF-α was also emphasized by Sharma et al. and Xie et al., who observed that levels of such cytokines were significantly increased in PE patients compared to healthy controls [57,58]. Furthermore, Laskowska et al., in agreement with us, indicated that TNF-α increased in PE patients and suggested this cytokine may play a role in the disease’s pathogenesis and consequences [59].

Saito et al., in an additional investigation, noted increased production of IL-2, interferon (IFN)-γ, and TNF-α by peripheral blood mononuclear cells (PBMCs) in women suffering from preeclampsia, along with significant associations between mean blood pressure and Th1 cytokine levels [60].

In our review we focused on the cytokines IL-15, IL-16, IL-17, and IL-35 and their levels in the serum of PE women at the time of the diagnosis in comparison to those of gestational age-matched controls. All of them have been implied to have a role in a successful pregnancy [15,16,17,21]. The significant role of IL-15 in poor pregnancy outcomes was previously reported by Sones et al. The authors examined how IL-15 activates NK cells and how these cells secrete IFN-γ, both of which are crucial for placental development [61]. In another, IL-17 was related to spontaneous miscarriages [62].

In this review, out of eight studies that analyzed the serum IL-15 levels in PE women, five studies [22,23,24,25,26] reported significant elevated levels of this cytokine in the PE group compared to the controls. Nevertheless, Lu et al. and Martinez-Fierro et al. noticed no significant difference [27,28]. In alignment with them was also Jonsson et al. However, none of the studies revealed decreased levels of IL-15 in the PE group [29]. This result highlights the importance of conducting more research to unveil the role of IL-15. This part of the study has great perspectives, as the research could be extended to other parameters, such as the study of IL-15 levels in the placenta or amniotic fluid of under-studied populations. Agarwal et al. investigated the levels of IL-15 in the placenta of PE women versus the normotensive ones and they revealed increased levels of IL-15 in the PE group [63].

IL-16 is a cytokine actively involved in the inflammatory response, and its role in conditions like preterm birth or premature rupture of membranes (PROM) has been the objective of research. Hsu et al. and Athayde et al. revealed increased levels of IL-16 in the amniotic cavity in women who experienced PROM [64,65]. On the other hand, Gallo et al. highlighted the elevated IL-16 levels in women who experienced uterine atony and postpartum hemorrhage (PPH). However, regarding IL-16’s role in PE, little is known [66]. All of the studies [22,24,26,27,30,31,32,33] included in this review concluded that there were statistically increased serum levels of the IL-16 in the PE group. Gu et al. made a critical finding that the severity of the condition was strongly correlated with the levels of IL-16 [31]. In alignment with them were also the studies by Hu et al., Radulescu et al., and Chaiworapongsa et al. [24,26,30]. This suggests that IL-16 plays a role in both the diagnosis and the follow-up. In addition, El-Barabie et al. not only spotted the correlation of the IL-16 levels with the severity of PE but also revealed a correlation with β-hCG levels [22].

The majority of the studies (sixteen out of nineteen) concluded a significant elevation of IL-17 in PE women compared to controls [26,34,35,36,37,39,41,42,43,44,45,46,47,48,49,50]. Only the study by Ozkan et al. observed decreased levels of IL-17 in the PE group, whereas two studies did not observe statistical difference [29,38,40]. The elevation of IL-17 levels during pregnancies complicated by PE was detected in most studies included in this review. This result is corroborated by research from Santner-Nanan et al., who indicated a decrease in Treg cell numbers alongside an increase in Th17 cell populations in placental complications of pregnancy [67]. In alignment with these results, was a metanalysis performed by Plug et al., who reported that the excessive increase in IL-17 was related to PE [68]. It appears that a slight increase in IL-17 is necessary to initiate and maintain a pregnancy, but excessive amounts of this cytokine are linked to complications during pregnancy, like PE. Well-balanced IL-17 levels are important to establish a healthy pregnancy, and maybe this explains the decreased levels of IL-17 that were observed in the Ozkan et al. study in the PE group [38]. Additionally, the study by Martínez-García et al. observed a little elevation in maternal plasma levels of IL-17 throughout the third trimester of uncomplicated pregnancies. The scientists ascribed the elevation of proinflammatory cytokine release near the delivery period to cervical dilatation and labor progression [69]. In Hee et al.’s prospective study, decreased levels of IL-17 were correlated with PROM and preterm birth, unveiling the importance of well-balanced levels of IL-17 in order to achieve an uncomplicated pregnancy [70]. However, Lang et al., in their prospective study, which included a PE group (*n* = 115), healthy pregnant women (*n* = 102), and a non-pregnant healthy population (*n* = 150), observed comparable maternal plasma levels of IL-17 between non-pregnant and healthy pregnant women [46].

On the other hand, IL-35 is a newly identified immunosuppressive cytokine exclusively produced by Treg cells, whereas recently, it has been shown that trophoblast cells continuously produce IL-35 during normal gestation [52]. It has been demonstrated that lymphocytes are naturally polarized towards IL-17 production, which results in decreased IL-35 levels. This shift disrupts the balance between proinflammatory (IL-17) and anti-inflammatory (IL-35) factors, especially in conditions like PE. In this review almost all of the studies revealed increased levels of IL-17 in PE groups; consequently, based on the above, decreased levels of IL-35 are expected, respectively. Indeed, among seven studies that investigated the role of IL-35, in five [35,36,37,38,51] the results were statistically decreased levels of IL-35 in the serum of PE women. Only in one study by Batebi et al., were increased levels of IL-35 in the PE group announced, and Agha et al. did not observe statistical differences between the two groups [40,50].

These results suggest that systemic inflammation and endothelial dysfunction could play a role in the development of PE. The relationship between preeclampsia and the overactive inflammatory response has been established; however, there is still a significant amount of room for additional research. In advance, all potential causes of inflammation during pregnancy must be thoroughly investigated, such as endometritis and viral infections like SARS-CoV-2, which have been demonstrated to be associated with pregnancy complications [71,72]. A very promising area of research is the extension of research to other interleukins, like IL-23 and IL-27. Another aspect of the research could focus not only on women’s blood but also on tracing interleukins in the placenta and the amniotic fluid. A further dimension of future investigations may involve the integrated study of tissue and serum biomarkers.

The imperative for additional exploration of the correlation between IL-27 and PE is evident, given the limited yet promising published evidence available. Coulomb-L’Herminé et al. discovered that genes encoding IL-27 (EBI3 and p28) and its receptor (IL-27R and gp130) are expressed in the placenta at different stages of pregnancy. These components are vital to the cytokine network that regulates local immune responses and promotes angiogenesis during human gestation [73]. Chen et al. observed that serum IL-27 levels were significantly elevated in women with PE, with the highest concentrations found in severe cases. IL-27 mRNA and protein expression were upregulated in placental tissues. The levels of the interleukin positively correlated with both systolic and diastolic blood pressure, suggesting a potential link between IL-27 expression and the severity of PE [74]. In parallel, Yin et al. found that the IL-27 receptor was overexpressed in placentas with PE, with a presence of TNF-α indicating a synergistic proinflammatory response [75].

Controversial and limited data were retrieved for the levels of IL-23 in PE, reflecting the necessity for further research. Poordast et al. reported lower levels in preeclamptic women compared to normotensive ones, while Gharesi-Fard et al. showed no statistical difference [41,76]. On the other hand, a case–control study by Forghani et al. pointed out an upward correlation between IL-23 levels and preeclampsia (PE), particularly in severe and early-onset cases, indicating the possibility of involving this cytokine in the pathogenesis of PE [77]. The role of interleukins, like IL-23 and IL-27, warrants further investigation, but this exceeds the scope of this review.

Further studies with large groups are required to investigate more the link between systemic inflammation and endothelial dysfunction with the mechanisms of PE.

This review presents some limitations. First of all, multiple pregnancies were excluded, and in addition, the PE group has been assessed without distinguishing the grades. It is assumed that the outcomes may be different between mild and severe PE. Furthermore, the varied design methodologies and the absence of geographical limitations augment the variability of the selected research.

## 5. Conclusions

This review emphasizes the substantial contribution of interleukins IL-15, IL-16, IL-17, and IL-35 to the immunopathology of preeclampsia. A well-controlled balance seems to be required for a successful pregnancy, but an exaggerated response is related to poor pregnancy outcomes. Most studies indicated that preeclamptic women exhibited increased serum concentrations of IL-15, IL-16, and IL-17 relative to normotensive controls. In contrast, IL-35, an immunosuppressive cytokine, was reduced, which implies that there is an imbalance between pro- and anti-inflammatory pathways that may contribute to the progression of the diseases.

These results substantiate the potential of these cytokines to serve as diagnostic and prognostic biomarkers in preeclampsia. Integrating cytokine assessment into clinical practice could enable earlier detection of women at risk, closer monitoring, and timely intervention to prevent PE-related severe complications. Finally, incorporating cytokine screening into prenatal care programs could completely affect public health, improving maternal outcomes, particularly in high-risk populations. Future research expanding to other cytokines, placental, and amniotic fluid markers may help establish reliable biomarker panels for early diagnosis and preventive strategies.

## Figures and Tables

**Table 1:** Ottawa Scale Newcastle Ottawa Assessment Scale.

	Selection				Compatibility	Outcome			Total
	Representativeness of the Exposed Cohort	Selection of the Non- Exposed Cohort	Ascertainment of Exposure	Outcome of Interest Not Present at Start of Study		Assessment of Outcome	Adequacy of Duration of Follow-Up	Adequacy of Completeness of Follow-Up	
El-Barabie et al., 2009 [22]	√	-	√	√	√	√	√	√	7/9
Mansouri et. 2007 [23]	√	-	√	√	-	√	√	√	6/9
Hu et al., 2007 [24]	√	-	√	√	√	√	√	√	7/9
Kalantar et al., 2013 [25]	√	-	√	√	-	√	√	√	6/9
Chaiworapongsa et al., 2024 [26]	√	√	√	√	√√	√	√	√	9/9
Lu et al., 2007 [27]	√	-	√	√	√	√	√	√	7/9
Martinez-Fierro et al., 2014 [28]	√	√	√	√	√	√	√	√	8/9
Jonsson et al., 2006 [29]	√	√	-	√	-	√	√	√	6/9
Rădulescu et al., 2016 [30]	√	√	√	√	√	√	√	√	8/9
Gu et al., 2008 [31]	√	√	√	√	√	√	√	√	8/9
Li W et al., 2017 [32]	√	-	√	√	√	√	√	√	7/9
Wang et al., 2006 [33]	√	√	√	√	-	√	√	√	7/9
Molvarec et al., 2015 [34]	√	√	√	√	√	√	√	√	8/9
Lu et al., 2020 [35]	√	√	√	√	√	√	√	√	8/9
Cao et al., 2015 [36]	√	√	-	√	-	√	√	√	6/9
Cao et al., 2018 [37]	√	√	-	√	-	√	√	√	6/9
Ozkan ZS et al., 2014 [38]	√	√	-	√	-	√	√	√	6/9
El Shahaway AA et al., 2019 [39]	√	√	-	√	-	√	√	√	6/9
Batebi et al., 2019 [40]	√	√	-	√	√	√	√	√	7/9
Poordast et al., 2017 [41]	√	√	-	√	-	√	√	√	6/9
Darmochwal-Kolarz D et al., 2012 [42]	√	√	-	√	-	√	√	√	6/9
Darmochwal-Kolarz D et al., 2017 [43]	√	√	-	√	-	√	√	√	6/9
Yang et al., 2017 [44]	√	√	-	√	-	√	√	√	6/9
Barnie et al., 2015 [45]	√	√	-	√	-	√	√	√	6/9
Lang et al., 2021 [46]	√	√	-	√	-	√	√	√	6/9
Perucci et al., 2023 [47]	√	√	-	√	-	√	√	√	6/9
Chen et al., 2018 [48]	√	√	-	√	-	√	√	√	6/9
Toldi et al., 2011 [49]	√	√	-	√	-	√	√	√	6/9
Agha et al., 2020 [50]	√	√	√	√	-	√	√	-	6/9
Li et al., 2020 [51]	√	√	√	√	-	√	√	-	6/9

**Table 2:** Studies included in the review regarding the interleukin (IL)-15.

Authors	Type of Study	Study Center	Recruitment Period	Ν Preeclampsia	N Control Group	Outcomes
El-Barabie et al., 2009 [22]	Observational	Egypt	12/2006– 9/2007	32	35	↑ IL-15 and β-hCG in preeclampsia (PE) Correlation with the severity of PE and with β-hCG
Mansouri et al., 2007 [23]	Observational	Iran	3–10/2006	30 (10 severe and 20 mild PE)	30	↑ IL-15 levels in PE group other: ↑ IL-12p70, IL-18, IL-4, and IFN-γ in PE group
Hu et al., 2007 [24]	Observational	China	N/A	37 (22 severe and 15 mild PE)	36	↑ IL-15 levels in PE group, correlation with the severity of PE
Kalantar et al., 2013 [25]	Observational	Iran	N/A	44	40	↑ IL-15 levels in PE group ↑ TNF-α in PE group
Chaiworapongsa et al., 2024 [26]	Case–control	USA	2006– 2010	96	213	↑ IL-15 in PE group Other: ↑ IL-6, IL-8, IL-12/IL-23p40, IL-16 with abnormal angiogenic profile with worse clinical severity Women with normal angiogenic profiles had only mild inflammatory response with ↑ MCP-4 and ↓ IL-1a
Lu et al., 2007 [27]	Retrospective	China	N/A	40	40	No significant difference
Martinez-Fierro et al., 2014 [28]	Observational	Mexico	2009– 2010	108 (48 severe and 60 mild PE)	69	IL-15 and IL-6 gene expression in PBMCs was undetectable or extremely low in both groups
Jonsson et al., 2006 [29]	Case–control	Sweden	N/A	15	15	No statistical difference
Total				402	478	

**Table 3:** Studies included in the review regarding the interleukin (IL)-16.

Authors	Type of Study	Study Center	Recruitment Period	Ν Preeclampsia	N Control Group	Outcomes
El-Barabie et al., 2009 [22]	Observational	Egypt	12/2006- 9/2007	32	35	↑ IL-16 in PE group Correlation with the severity of PE and β-hCG
Hu et al., 2007 [24]	Observational	China	N/A	37 (22 severe and 15 mild PE)	36	↑ IL-16 in PE group, Correlation with the severity of PE
Lu et al., 2007 [27]	Retrospective	China	N/A	40	40	↑ IL-16 in women who later developed PE
Radulescu et al., 2016 [30]	Prospective	Romania	1/2014– 7/2015	47	21	↑ IL-16 in PE group Correlation with the severity of PE
Chaiworapongsa et al., 2024 [26]	Case–control	USA	2006– 2010	96	213	↑ IL-16 Other: ↑ IL-6, IL-8, IL-12/IL-23p40, IL-15 with abnormal angiogenic profile with worse clinical severity Women with normal angiogenic profiles had only mild inflammatory response with ↑ MCP-4 and ↓ IL-1a
Gu et al., 2008 [31]	Observational	USA	N/A	66 (51 severe and 11 mild P)	63	↑ IL-16 in PE group Correlation with the severity of PE
Li W et al., 2017 [32]	Case–control	China	1/2014– 6/2016	131	65	Significantly higher
Wang et al., 2006 [33]	Observational	USA	N/A	34	37	Significantly higher
Total				510	479	

**Table 4:** Studies included in the review regarding the interleukin (IL)-35.

Authors	Type of Study	Study Center	Recruitment Period	N Preeclampsia	N Control Group	Outcomes
Li et al., 2020 [51]	Prospective	China	6/2016– 10/2017	24	24	↓ IL-35 levels in PE patients iTr35 cells significantly reduced in PE patients and positively correlated with IL-35 levels
Cao et al., 2015 [36]	Observational	China	6/2013–10/2014	44 (22 mild and 20 severe PE)	22	Decreased
Cao et al., 2018 [37]	Observational	China	1/2013–6/2016	33	33	Decreased
Batebi et al., 2019 [40]	Observational	Iran	N/A	100	100	↑ IL-35 in PE group Higher levels in severe PE vs. in mild PE and control
Lu et al., 2020 [35]	Observational	China	7/2015–7/2017	90	45	Significantly lower in PE patients than in healthy pregnant women
Ozkan ZS et al., 2014 [38]	Case–control	Turkey	8/2011–8/2012	40	40	Significantly lower
Agha et al., 2020 [50]	Case–control	Egypt	N/A	25	15	No difference
Total				354	279	

**Table 5:** Studies included in the review regarding the interleukin (IL)-17.

Authors	Type of Study	Study Center	Recruitment Period	N Preeclampsia	N Control Group	Outcomes
Molvarec et al., 2015 [34]	Observational	Hungary	2013– 2015	59	60	↑ IL-17A levels in PE women No correlation between il-17A and sFlt-1, PIGF, or sFlt-1/PIGF ratio IL-17A and sFlt-1/PIGF ratio had an additive effect on PE risk
Lu et al., 2020 [35]	Observational	China	7/2015–7/2017	90	45	↑ IL-17 in PE group
Cao et al., 2015 [36]	Observational	China	6/2013–10/2014	44 (22 mild and 20 severe PE)	22	↑ IL-17A in PE groups IL-17 levels positively correlated with DLL4 expression and negatively with Jagged-2
Cao et al., 2018 [37]	Observational	China	1/2013–6/2016	33	33	↑ IL-17 in PE group
Chaiworapongsa et al., 2024 [26]	Nested-case–control	USA	2006– 2010	96	213	↑ IL-17A in PE group
Ozkan ZS et al., 2014 [38]	Case–control	Turkey	8/2011–8/2012	40	40	Significantly lower in PE group
Jonsson et al., 2006 [29]	Case–control	Sweden	N/A	15	15	No statistical difference
El Shahaway et al., 2019 [39]	Prospective case–control	Egypt	N/A	20	20	Mean value of IL-17 in the PE group: 18.5 pg/mL, versus the control group: 4.3 pg/mL.
Batebi et al., 2019 [40]	Observational	Iran	N/A	100	100	No significant difference in IL-17A levels between PE and control group
Poordast et al., 2017 [41]	Observational case–control	Iran	N/A	30	30	Significantly higher in PE group
Darmochwal-Kolarz D et al., 2012 [42]	Case–control	Poland	N/A	34	27	IL-17A was significantly higher in PE group
Darmochwal-Kolarz D et al., 2017 [43]	Case–control	Poland	N/A	34	35	Significantly higher in PE group
Yang X et al., 2017 [44]	Case–control	China	N/A	60	20	Significantly higher in PE group
Barnie et al., 2015 [45]	Case–control	China	8/2013– 11/2014	17	17	Significantly higher in PE group
Lang et al., 2021 [46]	Case–control	China	3/2017–12/2019	115	102	IL-17A significantly higher in PE group
Perucci et al., 2023 [47]	Case–control	Brazil	NA	24	34	IL-17A significantly higher in PE group
Chen et al., 2018 [48]	Case–control	China	7/2016–3/2017	29	27	Significantly higher in PE group
Toldi et al., 2011 [49]	Case–control	Hungary	NA	20	22	Significantly higher in PE group
Agha et al., 2020 [50]	Case–control	Egypt	N/A	25	15	Significantly higher in PE group
Total				883	879	

## Data Availability

Not applicable.
